# Rehabilitation intervention to improve Recovery after an Episode of Delirium in adults over 65 years (RecoverED): a multicentre, single-arm feasibility study in NHS acute hospitals in the UK

**DOI:** 10.1136/bmjopen-2025-102316

**Published:** 2026-04-22

**Authors:** Louise Allan, Jinpil Um, Abby O’Connell, Shruti Raghuraman, Alison Bingham, Abigail Laverick, Kirstie Chandler, James Connors, Aseel Mahmoud, Annie Hawton, Elizabeth Goodwin, Sarah Morgan-Trimmer, Victoria A Goodwin, Obioha C Ukoumunne, Thomas A Jackson, Sarah J Richardson, Lesley Collier, Jon Glasby, Bethany Whale, Phoebe Dawe, Naomi Burnett-Fry, Linda Clare, on behalf of the RecoverED team

**Affiliations:** 1 Medical School, University of Exeter Faculty of Health and Life Sciences, Exeter, UK; 2 NIHR Applied Research Collaboration South West Peninsula, University of Exeter, Exeter, UK; 3 Our Future Health, Manchester, UK; 4 Exeter Clinical Trials Unit, University of Exeter, Exeter, UK; 5 Institute of Inflammation and Ageing, University of Birmingham, Birmingham, UK; 6 AGE Research Group, NIHR Newcastle Biomedical Research Centre, Newcastle University Faculty of Medical Sciences, Newcastle upon Tyne, UK; 7 School of Social Policy and Society, University of Birmingham, Birmingham, UK; 8 University of Exeter Faculty of Health and Life Sciences, Exeter, UK

**Keywords:** Delirium, Dementia, Clinical trials, Feasibility Studies

## Abstract

**Objectives:**

To test a theory-informed, person-centred rehabilitation intervention for older adults following a hospital admission complicated by delirium, developed in line with the Medical Research Council framework for complex interventions, to determine whether: (a) the intervention is acceptable to individuals with delirium and (b) a definitive trial and parallel economic evaluation of the intervention are feasible.

**Design:**

Multicentre, single-arm feasibility study.

**Participants:**

19 patient (aged >65 years old) and carer pairs were recruited from six National Health Service acute hospitals across the UK.

**Intervention:**

Home-based rehabilitation programme designed to support recovery after hospital discharge, addressing cognitive, physical, physiological and psychosocial needs. Delivered by a trained team of occupational therapists, physiotherapists and rehabilitation support workers, the intervention included a comprehensive home assessment, collaborative goal setting, up to 10 personalised sessions over 12 weeks and the use of a recovery record to guide progress, education and psychosocial support.

**Outcome measures:**

Examined aspects of feasibility including eligibility, recruitment, data collection, attrition, acceptability of the rehabilitation intervention and potential to calculate cost-effectiveness.

**Results:**

In total, 419 patients were identified as having delirium and 36 met the full eligibility. 19 patient and carer pairs agreed to participate in the study (consent rate 53%; 95% CI 35% to 70%) with 13 participants going on to start the intervention (68%; 95% CI 43% to 87%) and 10 participants completing final follow-up (53%; 95% CI 29% to 76%). Baseline assessments were conducted either during hospitalisation or postdischarge, with initial assessments occurring a mean of 18 days (SD=13.0) postdischarge, and 77% completed within 14 days. Participants completed a mean of eight sessions (SD=2.9). 19 participants completed the primary outcome at baseline, while 10 participants completed it at 6-month follow-up. The economic evaluation indicated a total cost of £1249.29 per participant, covering assessments, intervention sessions and training costs.

**Conclusions:**

The intervention showed feasibility among older adults recovering from delirium, as evidenced by the trial processes for participants who entered the study. However, recruitment challenges indicate a need for better strategies and further research through a definitive randomised controlled trial to demonstrate the effectiveness and cost-effectiveness of the intervention.

**Trial registration number:**

ISRCTN15676570

**STRENGTHS AND LIMITATIONS OF THIS STUDY**

- Comprehensive, person-centred approach, tailoring the intervention to individual cognitive, physical, physiological and psychosocial needs.
- The study used a recovery record to support goal setting, progress tracking, education and psychosocial support.
- Embedded process and economic evaluation to assess feasibility and cost-effectiveness for a future randomised controlled trial.
- Recruitment and retention challenges, impacting sample size and generalisability.
- Single-arm design precluding direct comparison of the intervention with standard care.

## Introduction

 Delirium, a complex acute cognitive disorder, poses significant challenges in the healthcare landscape, particularly among hospitalised older people. The main characteristic involves disruptions in attention and consciousness, alongside cognitive deficits and alterations in behaviour. Individuals experiencing delirium may exhibit symptoms such as restlessness, withdrawal and agitation, often accompanied by visual hallucinations and delusions. Delirium occurs as a direct physiological consequence of an underlying medical condition and typically manifests with a sudden onset and variable course.^1^

The profound impact of delirium extends beyond the individual, encompassing family caregivers and imposing substantial burdens on healthcare systems. Extensive research has underscored the association between delirium and adverse outcomes, including prolonged hospital stays, increased healthcare costs, cognitive and functional decline and elevated mortality rates.^2^,^9^ Moreover, the communication barriers inherent in delirium exacerbate caregiver distress and strain interpersonal relationships, highlighting the pressing need for comprehensive support structures.^10^,^13^

Besides being significantly distressing for individuals with delirium and their caregivers, delirium imposes a substantial financial burden, with total inpatient costs per episode ranging from £12 575 to £49 689.^14^ Furthermore, cognitive and functional deficits stemming from delirium can endure for months postepisode, with a notable proportion of individuals failing to fully recover, as evidenced by a 21% persistence rate of delirium after 6 months.^15^ Delirium also increases the risk of long-term cognitive decline, dementia and functional deterioration,^39 11 16^,^19^ often leading to a greater need for care or institutionalisation.^5 20 21^ Research indicates that functional impairment may persist for up to 1 year.^22^,^25^ Studies illustrating a decline in performance of activities of daily living (ADL) postdelirium underscore the ongoing care needs of affected individuals, which could potentially be alleviated through rehabilitation efforts.^2 26^

Despite growing recognition of the significance of delirium, there remains a notable gap in research focusing on the post-hospitalisation phase and the support needs of affected individuals and their caregivers.^27^ This gap is particularly problematic because while acute-phase management has advanced, postdischarge care often remains fragmented. Consequently, patients and their families are frequently left to navigate the long-term cognitive and functional consequences of delirium, which limited structured support, increasing the risk of re-hospitalisation and caregiver burnout. The RecoverED (Recovery after an Episode of Delirium) programme aimed to address this gap by developing a theory-based, novel, multicomponent complex intervention to support recovery in older people following an episode of delirium.^28^ This paper reports findings from a feasibility study designed to determine whether a definitive randomised controlled trial (RCT), including a full economic evaluation, is achievable. An embedded process evaluation was conducted to examine the implementation of the intervention, including its acceptability and feasibility for participants. The study also assessed the feasibility of collecting data to inform the selection of primary and secondary outcome measures for a definitive RCT. These findings were intended to facilitate the iterative refinement and the intervention prior to fully scale testing.

## Methods

### Design

This study was a multicentre, single-arm feasibility study of a home-based rehabilitation intervention, incorporating embedded process and economic evaluations. The study employed a mixed-methods design combining quantitative and qualitative approaches. This design was chosen because feasibility testing requires both numerical data on recruitment and retention, and explanatory qualitative depth to understand participant and staff experiences. Implementation fidelity and acceptability were examined through an embedded mixed-methods process evaluation, drawing on data collected from multiple stakeholders between June 2023 and July 2024 to capture diverse perspectives.

The study was developed with substantial input from a patient and public involvement and engagement (PPIE) group, whose contributions were integral to shaping the intervention, refining recruitment strategies and ensuring the relevance of outcome measures. The protocol for this study, including details of the intervention, recruitment strategies and assessment measures, has been outlined previously.^29^ Changes to the study protocol are presented in the online supplemental additional file 1. The study design and reporting were guided by the Consolidated Standards of Reporting Trials (CONSORT) 2010 extension statement to randomised pilot and feasibility trials.^30^

### Setting

Participants were recruited from six National Health Service (NHS) hospitals across the UK, located in Birmingham, Devon, Edinburgh, London, Newcastle and Nottingham. All participating sites were university teaching hospitals, representing large tertiary care environments that serve complex and diverse patient populations in different regional contexts. The intervention was administered in the participants’ own homes postdischarge from an acute admission. Depending on the site’s organisational structure, the acute hospital either provided the community therapy services directly or collaborated with a separate community provider for intervention delivery. Follow-up assessments were conducted either in the participants’ homes or at the NHS site depending on the participants’ preferences and abilities, and the capacity of the local research delivery team.

### Eligibility

Participants eligible for the study were aged over 65, identified as having delirium during their hospital stay through routine clinical care, typically following a positive delirium screen (such as the 4AT or Confusion Assessment Method (CAM)^31^) and confirmation of a clinical diagnosis by the treating clinical team, and expected to return to a private dwelling after discharge or within 4 weeks of intermediate care. They also needed a carer who could participate in the study. Those for whom a diagnosis of delirium could not be confirmed, those receiving palliative care, those unable to communicate due to advanced dementia or aphasia, and any who were involved in other intervention studies were excluded. Carers had to be family members or friends in regular contact (at least 1 hour per week), able to communicate in English for proxy measures, and capable of providing informed consent. There were no exclusion criteria for carers.

### Recruitment

Participants were recruited from in-patient wards. As part of standard care, patients were screened for delirium using well-established methods such as the CAM or the 4AT Rapid Clinical Test,^31^ with screening undertaken by members of the clinical care team (including nursing and healthcare professionals) as part of routine ward practice. Patients who screened positive were discussed with a clinical researcher embedded within the clinical team, in consultation with ward clinicians, to establish whether a clinical diagnosis of delirium had been made. It is important to note that there was variability across the six participating sites in how routinely and systematically delirium screening tools were applied in clinical practice, which influenced the number of patients identified and referred for eligibility assessment.

### Intervention

The intervention aimed to support at-home recovery for individuals posthospital discharge through a structured rehabilitation programme. The intervention was developed using a realist-informed approach aligned to the Medical Research Council framework for complex interventions, underpinned by an explicit programme theory and logic model that identified interrelated physical, cognitive and psychosocial mechanisms to support recovery following delirium.^28 32^ This programme was tailored to fit the unique needs of each participant, involving a comprehensive set of activities that addressed cognitive, physical, physiological and psychosocial recovery. Initiated within 2 weeks of hospital discharge, a community physiotherapist (PT) and occupational therapist (OT) conducted a comprehensive home assessment and medication review and engaged in goal setting. Goal setting was a collaborative process involving the participant and their carer, ensuring that goals were aligned with their priorities and needs. These goals, established during the initial visit, focused on enhancing recovery and independence.

The home assessment included evaluating existing care packages, home safety, functional ability and mobility status, and providing advice on accessing community resources. Following the assessment, a rehabilitation support worker (RSW) delivered up to 10 personalised sessions over 12 weeks in the home setting, guided by a manual. A mid-intervention review by the OT and/or PT ensured that the intervention remained aligned with the participant’s goals. Throughout the intervention, participants used a recovery record to document their progress and plan activities. Additionally, the recovery record housed the advice and education elements of the intervention, including strategies to support physical, cognitive and emotional recovery. It also incorporated psychosocial interventions, offering tailored support to enhance well-being and promote engagement in meaningful activities.

The intervention team, comprising OTs, PTs and RSWs, underwent a thorough training programme developed by the study team. The training programme, informed by the theoretical framework of the intervention, covered key knowledge and skills including delirium and recovery education, principles of personalised goal setting, delivery of physical, cognitive and psychosocial rehabilitation components, use of the recovery record, safeguarding and escalation procedures, and working collaboratively with carers. This programme, designed for flexibility and accessibility, was delivered through bite-sized modules totalling approximately 4.5 hours, complemented by additional reading of the manual. The training also included virtual peer support sessions every 3 weeks, ensuring continuous support and improvement.

### Feasibility and acceptability

Feasibility outcomes in this study included various indicators to assess the practicality of implementing the intervention. Table 1 summarises the feasibility outcomes assessed in this study. Specific measures included the identification of individuals with delirium, participants’ eligibility, recruitment rates and retention throughout the study duration.

**Table 1:** Feasibility objectives matched to outcomes

Feasibility objectives	Outcome measures	Data collector	Time point(s) of evaluation of this outcome measure
Primary objective			
Objective A: the primary objective is to conduct a feasibility study of the rehabilitation intervention in older adults who have had delirium to determine if the intervention is acceptable to patients and their carers.	The proportion of eligible people with delirium who agree to participate in the study*	Clinical researcher	Recruitment
	The proportion of carers who agree to participate in the study	Clinical researcher	Recruitment
	The acceptability of the intervention assessed during the process evaluation, using semistructured qualitative interviews with participants, carers and intervention staff	Qualitative researcher	Postintervention
	The accuracy of the sample size calculation for the definitive RCT	Research team/statistician	Throughout
Secondary objectives			
Objective B: to examine the acceptability of the intervention for underserved populations via a process evaluation	The acceptability of the intervention assessed during the process evaluation	Qualitative researcher	Postintervention
Objective C: to test the ability to collect the data required to address the primary and secondary outcomes for the definitive randomised controlled trial (RCT)	The number of people with delirium identified on hospital wards	Clinical team/clinical researcher	Recruitment
	The proportion (and number) of people with delirium who meet the eligibility criteria	Clinical team/clinical researcher	Recruitment
	The proportion of participating people with delirium who start the intervention	Rehabilitation staff (OT, PT or RSW)	3 months
	The proportion of participating people who complete ≥60% of the intervention sessions*	Rehabilitation staff (OT, PT or RSW)	3 months
	The proportion of participating people with delirium who remain in the study until final follow-up at 6 months*	Clinical researcher	6 months
	The proportion of people with delirium providing valid outcome data for each primary and secondary outcome measure (described below) at 3 and 6 months follow-ups	Clinical researcher	3 months and 6 months
	The estimated SD and 6 months follow-up rate for the proposed primary outcome, in order to either verify or inform revision of the proposed sample size calculation for the definitive RCT	Research team/statistician	6 months
Objective D: to test the cost-effectiveness framework for the definitive RCT	The proportion of people with delirium providing valid outcome data for each primary and secondary outcome measure (described below) at 3 and 6 months follow-ups	Clinical researcher/health economist	3 months and 6 months
Objective E: to perform iterative refinement of the intervention for the definitive RCT	The acceptability of the intervention assessed during the process evaluation*	Qualitative researcher	Postintervention

* Feasibility outcomes marked with an asterisk were used to determine whether the study me the criteria for progression to the definitive RCT.

OT, occupational therapist; PT, physical therapist; RSW, rehabilitation support worker.

The progression criteria, detailed in table 2, were used to determine if the study met the criteria for advancing to a definitive RCT:

### Process evaluation

**Table 2:** Progression criteria for RCT

Domain	Proceed with RCT (green light)	Do not proceed (red light)
Recruitment	≥25% of eligible participants consenting (or consultee agreeing) to feasibility study	<10% of eligible participants consenting to feasibility study
Completion of intervention	≥70% participants attend ≥60% of sessions as planned	<30% participants attend ≥60% of sessions as planned
Retention	Retention of ≥60% of recruited participants for key outcome data at 6 months	Retention of <50% of consented participants for provision of key outcome data at 6 months
Intervention acceptability	Evidence from the process evaluation that the intervention can be delivered with fidelity and that it is acceptable to people vulnerable to health inequalities	Evidence from the process evaluation that the intervention cannot be delivered with fidelity and that it is not acceptable to people vulnerable to health inequalities

RCT, randomised controlled trial.

An embedded process evaluation was conducted to examine the acceptability and implementation fidelity of the novel RecoverED intervention. The process evaluation drew on multiple data sources, including case report forms (CRFs) completed by RSWs during each session, semi-structured interviews conducted with participants, carers and healthcare professionals, focus groups with the healthcare professional and training and supervision logs. Qualitative data were analysed using a deductive framework approach informed by a modified Conceptual Model for Implementation Fidelity,^33 34^ while quantitative data were summarised descriptively. Findings were integrated using a joint display table to support systematic comparison across data sources and provide a comprehensive account of intervention implementation.

### Assessments

Baseline assessments were conducted by trained researchers (research nurses or equivalent) after participants provided consent and while they remained in hospital. However, it is important to note that a change to the protocol allowed for baseline assessments to also take place postdischarge in the participants’ homes. These assessments encompassed a comprehensive range of participant-reported outcome measures and clinical evaluations. A detailed description of these measures can be found in the protocol paper.^29^

Medical history and demographic information (eg, age, sex, ethnicity) were collected. A detailed delirium assessment was completed by trained research nurses or equivalent using standardised Diagnostic and Statistical Manual of Mental Disorders (DSM)-5 criteria,^35^ with additional assessments including the Observational Scale of Level of Arousal^36^ and Memorial Delirium Assessment Scale scales.^37^

Follow-up assessments were scheduled at 3 and 6 months postdischarge, with a±2 week time frame to accommodate logistical considerations while ensuring data integrity. These included measures of cognitive function, aADL, quality of life and other relevant outcomes, administered through pseudonymised CRFs and questionnaires. Data collection was facilitated through the Research Electronic Data Capture platform, ensuring efficient and secure handling of study data throughout the assessment period.

### Outcome measures

The proposed primary outcome for a future definitive RCT was functional ability, assessed using the Disability Assessment for Dementia (DAD).^38^ The DAD measures basic and instrumental ADL that are of particular importance to older adults and has been recommended for use in dementia trials. This measure was selected on the basis that, although most participants in this study did not have a formal diagnosis of dementia, cognitive deficits—particularly affecting executive function—are common following delirium. An activity of daily living scale originally developed for dementia was therefore considered likely to be sensitive to change in this population. Given the limited evidence on the use of the DAD in people recovering from delirium without dementia, this feasibility study also aimed to assess the acceptability, completeness and responsiveness of the DAD to inform outcome selection for a future definitive trial.

Secondary outcomes assessed mobility, delirium persistence or recurrence, attention, arousal, cognition, mood, well-being, quality of life, carer outcomes and health and social care resource use. These measures were selected to capture the multidimensional impact of delirium and recovery and are described in detail in online supplemental additional file 2 and the published study protocol.^29^

### Statistical analysis

Descriptive statistics were used to summarise recruitment, retention and adherence rates. Continuous outcomes were presented as means and SDs, while categorical outcomes were presented as frequencies and percentages. No inferential statistical tests were conducted, as the primary aim of the study was to assess feasibility. The SDs of the outcomes were estimated to help inform the sample size calculation for a future definitive RCT.

### Sample size calculation

This study was a single-arm feasibility study, primarily focused on estimating feasibility parameters to guide the design of a future definitive RCT, rather than assessing the effectiveness of the intervention. The study aimed to recruit a total of 60 participants. Assuming that key feasibility parameters, such as the follow-up rate and the percentage of participants attending at least 60% of the intervention sessions, are 70%, the target sample size is large enough to estimate these parameters with a 95% CI ranging from 57% to 81%.

### Economic evaluation

The study aimed to assess the feasibility of conducting a full, policy-relevant, cost-effectiveness analysis (CEA) of the intervention alongside a future RCT. The objectives of the health economic analysis were:

1. to estimate the resource use and associated costs of delivering the intervention;
2. to test methods for collecting data on participant and carer resource use;
3. to test methods for collecting data on health economic outcomes: participant health-related quality of life (HRQL) using the EuroQol 5-Dimension 5-Level (EQ-5D-5L), EQ-5D-5L-Proxy-V2, Dementia Quality of Life - Utility (DEMQOL-U) and DEMQOL-Proxy-U, carer HRQL using the EQ-5D-5L,^39^ participant well-being using the ICECAP-0^40^ and carer well-being using the ICEpop CAPability measure for Adults (ICECAP-A)^41^;
4. to produce summary statistics describing resource use, quality-adjusted life-years (QALYs) and well-being-adjusted life-years (WALYs).

A bespoke resource use questionnaire (RUQ) was developed by the health economists, research team and PPIE group for the study, informed by recent guidance on items for inclusion in resource use measures.^42 43^ The RUQ was completed by carers at baseline and at 3 and 6 months follow-up, and included NHS and personal social services (PSS) resources, as well as broader societal resources, including other support services, informal care and out-of-pocket expenses.

Further information on the health economics methods used in this trial is presented in online supplemental additional file 3.

### Patient and Public Involvement

PPIE was central to the RecoverED programme. A PPIE group—consisting of individuals with lived experience of delirium and representatives from diverse backgrounds—was integrated into the study’s governance, serving on the expert panel, Trial Management Group and Programme Steering Committee. The group’s contributions spanned several key areas, including the refinement of participant information sheets to remove technical jargon and the review of rehabilitation manuals to ensure home-based exercises were practical. Additionally, PPIE members collaborated with health economists to refine the Health and Care RUQ and advised on qualitative interviewing and strategies to address language and cultural barriers for underserved populations. Furthermore, members engaged in detailed dialogues to balance clinical relevance with lived experience when selecting primary outcome measures for future trials. Finally, members met regularly to advise on study conduct and were involved in the interpretation and dissemination of the final study findings.

## Results

### Feasibility

**Figure 1:**
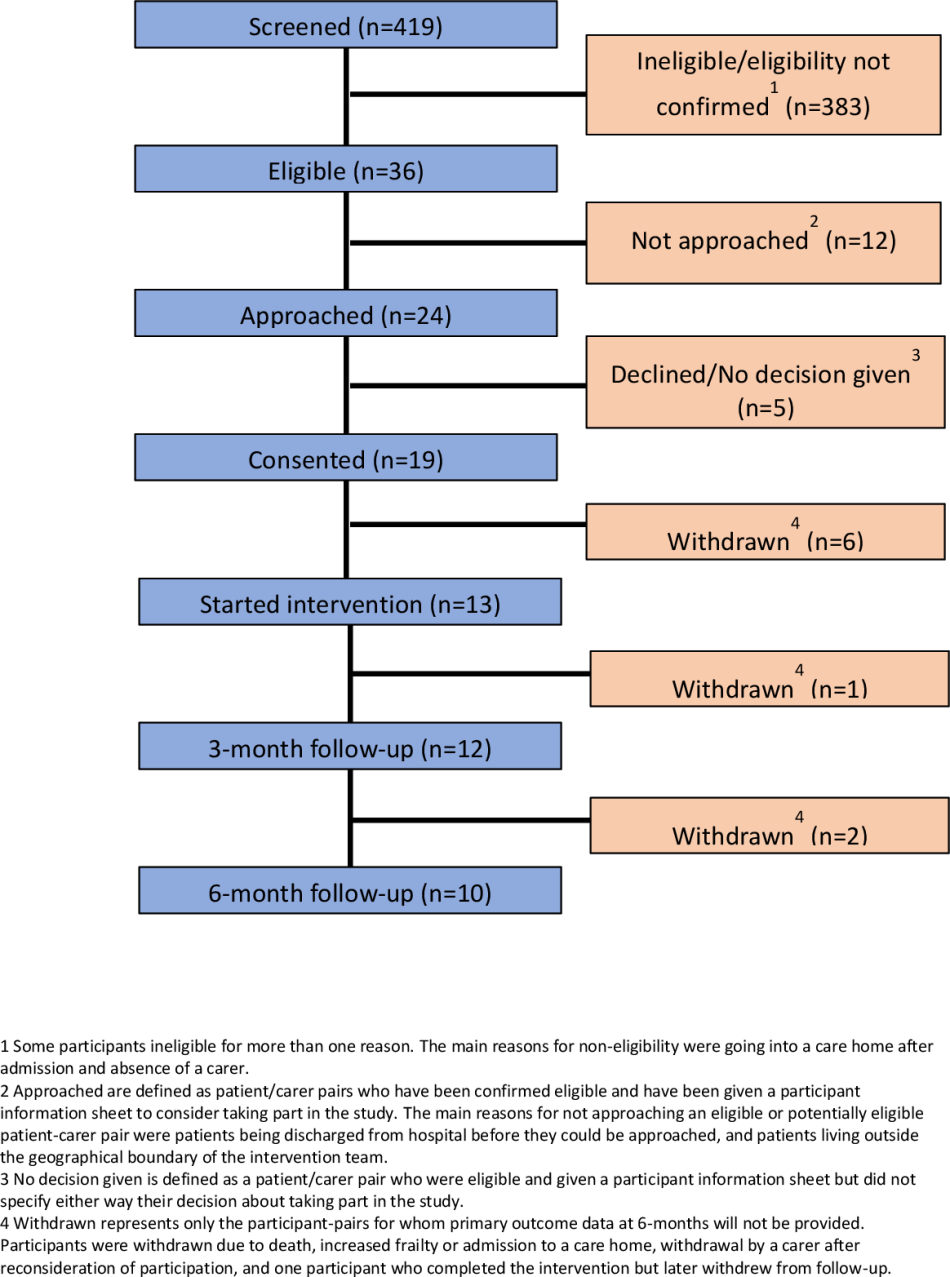
RecoverED flow diagram of participants. RecoverED, Recovery after an Episode of Delirium.

In total, 419 patients were identified across six hospitals as having delirium and 36 (9%; 95% CI 6% to 12%) met the full eligibility criteria for participation in the study (figure 1). 19 patient and carer pairs agreed to participate in the study (consent rate 53%; 95% CI 35% to 70%) with 13 participants going on to start the intervention (68%; 95% CI 43% to 87%) and 10 participants completing final follow-up (53%; 95% CI 29% to 76%) in the study (table 3).

**Table 3:** Summary of feasibility outcomes

Feasibility outcome measure	n/N	% (95% CI)
Number of people identified with delirium	435	
Proportion of people with delirium who meet eligibility criteria	36/435	9% (6% to 12%)
Proportion of eligible patient and carer pairs who agree to participate in the study	19/36	53% (35% to 70%)
Proportion of participating people with delirium who start the intervention	13/19	68% (43% to 87%)
The proportion of participating people who complete ≥60% of the intervention sessions	10/19	53% (29% to 76%)
The proportion of participants with delirium who remain in the study until final follow-up	10/19	53% (29% to 76%)

### Participant characteristics

All participants were white, the mean age was 83.8 years (range 73 to 97), and 12 were men and 7 were women. The mean clinical frailty score of the participants was 5.3 (SD 1.8); 5 participants (26%) had dementia as a co-morbidity (table 4). The carer participants were mostly women (15), and all carer participants were white. Carers had a mean age of 66.6 years (range 43 to 86) with 11 of the carers being a spouse or partner of the participant; 7 were children and 1 was a grandchild.

**Table 4:** Summary of key patient and carer demographics

Patient demographics	N	*	Carer demographics	N	*	Screening Demographics	N	*
Age, mean (SD)	19	83.8 (6.5)	Age, mean (SD)	18	66.6 (14.4)	Age, mean (SD)	435	85.0 (7.4)
Sex	19		Sex	19		Sex	435	
Male, n (%)		12 (63%)	Male, n (%)		4 (21%)	Male, n (%)		203 (47%)
Female, n (%)		7 (37%)	Female, n (%)		15 (79%)	Female, n (%)		232 (53%)
Ethnicity	19		Ethnicity			Ethnicity	424	
White, n (%)		19 (100%)	White, n (%)			White, n (%)		407 (96%)
Living arrangement	19		Relationship to patient	19		Asian/British Asian, n (%)		10 (2%)
Alone, n (%)		4 (21%)	Partner, n (%)		11 (58%)	Black/African/Caribbean		
Partner, n (%)		11 (58%)	Child, n (%)		7 (37%)	Black British, n (%)		5 (1%)
Other family, n (%)		4 (21%)	Grandchild, n (%)		1 (5%)	Other, n (%)		2 (1%)
Dementia diagnosis	19		Patient contact frequency	19				
Yes, n (%)		5 (26%)	Every day, n (%)		17 (90%)			
No, n (%)		14 (74%)	Most days, n (%)		1 (5%)			
			Once a week, n (%)		1 (5%)			

*Mean (SD) / N (%)

### Data completeness and clinical outcomes

Intervention adherence was defined as attending six or more of the intervention sessions over the duration of the study. This was achieved by 10 of the 19 participants (53%). Six-month follow-ups were completed within the ±2-week window for all 10 participants who remained in the study until the final follow-up (53% of the initial 19 participants).

All 19 participants completed the proposed primary outcome, the Disability Assessment for Dementia (DAD) at baseline with a mean (SD) of 43.7 (27.1). 12 participants went on to complete the DAD at 3 months with a mean (SD) of 53.5 (28.5). 10 participants completed the DAD at 6 months with a mean (SD) of 64.5 (27.9). An adapted DSM-5 algorithm assessment of delirium was completed based on the participant’s responses to questions throughout the trial. In this assessment, 15 of the 19 participants (79%) were identified as having sufficient markers for delirium at baseline, but no participants were identified as having sufficient markers at either follow-up.

### Feasibility of cost-effectiveness framework

#### Intervention resource use and costs

Data were successfully collected for most resources required for delivery of the intervention (direct staff contact time, travel time, mileage, training and materials). Data collection was unsuccessful for staff time spent on non-contact activities (session preparation, updating records, supervision); therefore, estimates for this component were provided by the research team. The mean cost per participant of the intervention was £1249. The resources required for delivery of the intervention and their associated costs are detailed in table 5.

**Table 5:** Resources required for delivery of the intervention and their associated costs

Session costs*	Source						Mean cost per participant
Initial assessment	Participant case report forms						£170.94
Intervention sessions	Participant case report forms						£705.95
Staff training and support	Source	AfC band	Hourly rate	Mean time per therapist (hrs)	Total cost per therapist	Mean time per participant (hrs)	Mean cost per participant§ ¶**
Non-contact time†	Estimate from study team	Band 4	£36.00			5.75	£207.00
Training (RSW)	Individual staff training logs	Band 4	£36.00	6.73	£242.14	0.06	£2.02
Training (OT)	Individual staff training logs	Band 6	£53.00	5.47	£290.08	0.09	£4.83
Training (Physio)	Individual staff training logs	Band 6	£53.00	5.47	£290.08	0.09	£4.83
Providing supervision	Estimate from Study Team	Band 6	£53.00			2.50	£132.50
Materials	Source	AfC band	Hourly rate	Staff time (hours)	Total staff cost	Total printing cost‡	Mean cost per participant¶ ††
Therapist manual	Estimate from study Team	Band 4	£36.00	0.25	£9.00	£30.24	£0.33
Participant record	Estimate from study team	Band 4	£36.00	0.17	£6.00	£14.88	£20.88
Total cost of RecoverED intervention per participant							£1249.29

* Includes contact time, travel time and mileage.

† Includes time spent planning sessions, completing paperwork and receiving supervision.

‡ Includes paper and printing costs.

§ Assumes that each participating dyad is supported by one RSW and either one OT or one physio.

¶ Assumes one RecoverED team per site (ie,1 RSW, 1 OT, 1 physio), able to support 60 participants per year.

** Assumes that staff remain in post for 2 years after receiving training.

†† Assumes a ‘lifetime’ for the manual of 2 years before it needs replacing.

Abb AfC, Agenda for Change; OT, occupational therapist; RecoverED, Recovery after an Episode of Delirium; RSW, rehabilitation support worker.

#### Resource use questionnaire

While rates of missing data for individual resource use items were moderate to good (most ranging from zero to two missing observations per item for the ten carers who provided data at 6-month follow-up, with three to four missing observations for out-of-pocket expenses), the items that were missing varied between participants. Only participants with complete data across all items, at both follow-up points, can be included in the calculation of total costs. As a result, when all items were combined to produce total costs across the 6-month follow-up period, total NHS/PSS costs were based on only three participants, reducing to two participants when societal costs were added. Responses to the RUQ at 6-month follow-up and disaggregated costs of resource use across the combined follow-up period are reported in online supplemental additional file 4.

#### QALY and WALY measures

Table 6 presents patient QALYs based on self-reported and proxy-reported EQ-5D-5L and DEMQOL, carer QALYs based on self-reported EQ-5D-5L, patient WALYs based on the ICECAP-O and carer WALYs based on the ICECAP-A. These figures indicate the number of participants providing complete data across all time-points (baseline, 3-month follow-up and 6-month follow-up).

**Table 6:** Quality-adjusted life-years and well-being-adjusted life-years (QALYs/WALYs) for patient and carer participants

	Obs	Mean	SD	Min	Max
QALYs					
Patient proxy-reported EQ-5D QALYs	10	0.227	0.173	−0.084	0.416
Patient self-report EQ-5D QALYs	8	0.273	0.168	−0.013	0.423
Carer EQ-5D QALYs	9	0.403	0.092	0.244	0.494
Patient proxy-reported DEMQOL QALYs	10	0.318	0.028	0.280	0.358
Patient self-report DEMQOL QALYs	9	0.296	0.031	0.248	0.339
WALYs					
Patient ICECAP-O WALYs	8	0.368	0.075	0.187	0.425
Carer ICECAP-A WALYs	9	0.427	0.056	0.347	0.500

DEMQOL, Dementia Quality of Life measure; EQ-5D, EuroQol 5-Dimension measure; ICECAP-A, ICEpop CAPability measure for Adults; ICECAP-O, ICEpop CAPability measure for Older people; Max, maximum; Min, minimum; Obs, observations.

### Intervention fidelity and acceptability

Overall, the intervention was delivered with good fidelity to its core functions and was well accepted by both participants and professionals. Fidelity was primarily demonstrated through person-centred tailoring, with healthcare professionals planning and adapting intervention components in line with participant-led goals. Psychosocial support was delivered more frequently than originally planned, reflecting its perceived importance in delirium recovery. While adherence to the intended dose was moderate, withdrawal was largely driven by deteriorating health or care transitions rather than dissatisfaction with the intervention. Among those who completed the programme, acceptability was high, with participants reporting meaningful benefits and professionals valuing the flexibility and relevance of the intervention. Together, these findings support the feasibility of the intervention and its readiness for further optimisation and testing within a definitive RCT. Detailed findings from this process evaluation will be reported in a separate paper.

## Discussion

The study revealed several important findings regarding the feasibility of recruiting participants in hospital diagnosed with delirium for a postdischarge intervention. Of those screened, only 9% met the eligibility criteria, reflecting challenges related to study design. The primary recruitment challenge was the lower-than-anticipated number of patients identified for screening. Among those who were eligible, a consent rate of 53% was achieved for patient and carer pairs, indicating a good level of willingness to participate. Of those who consented, 13 participants (68%) initiated the intervention, and 10 completed the final follow-up.

The clinical frailty score indicated that the sample comprised individuals with notable frailty, which is consistent with the expected profile of older adults with delirium. This level of frailty is likely to have contributed to the low retention rate observed at 6 months, given the increased risk of functional decline, rehospitalisation and mortality in this population. These findings highlight implications for the design of an RCT trial, suggesting that primary outcome assessment at earlier time points may be more feasible and appropriate for capturing intervention effects in a highly frail cohort.

Adherence to the intervention was moderate, with 53% of the participants attending six or more sessions. While this suggests that a substantial proportion of participants were able to engage with the intervention, this figure fell below the prespecified ‘green light’ progression criterion of ≥70% of participants attending ≥60% of sessions, as outlined in table 2. Similarly, retention to final follow-up did not meet the progression threshold, with 53% of recruited participants (10/19) remaining in the study and providing outcome data at 6 months, compared with the target of ≥60%. In contrast, evidence from the embedded process evaluation indicated that, among participants who remained engaged, the intervention was acceptable and could be delivered with fidelity. The primary outcome, assessed through the DAD, increased from a mean score of 43.7 at baseline to 64.5 at 6 months. This descriptive change cannot be attributed to the intervention in the absence of a control group.

Health economic analysis indicated that the mean cost per participant for the intervention was £1249, with comprehensive data collected on the resources utilised. However, data collection for certain non-contact activities proved challenging, emphasising a need to focus on how to collect this data in a future trial. In addition, there was various missing data on the use of health, social care and societal resources by participants and carers, suggesting a need to review processes for the collection of these data in a definitive trial.

While the study demonstrated the potential to recruit individuals with delirium once identified, significant challenges remained in reaching enough patients for screening. A number of factors contributed to this. Research nurse capacity to undertake screening was not as extensive as had been planned and costed for in the funding of the study. This was due to limitations in the infrastructure and staffing levels for undertaking research recruitment in the UK. The diagnosis of delirium was primarily made by clinical teams rather than research staff screening all inpatients for delirium. This revealed weaknesses in screening processes for delirium in the UK, a problem which is also reported internationally.^44^,^46^ Future studies may benefit from incorporating a more standardised screening strategy, such as delirium assessments conducted by trained research personnel at regular intervals (eg, twice daily) using validated tools. Such an approach may improve the consistency and fidelity of delirium detection in research settings and reduce the variability associated with reliance on routine clinical screening practices.

The National Institute for Health and Care Excellence (NICE)^47^ recommends using the 4AT to screen all older inpatients for delirium on admission, but this was not carried out routinely in several of the sites. This meant that the total number of people screened for this study was much lower than expected. It was expected that 1200 people would be screened over the course of 6 months but over an extended period of recruitment, only 419 were screened. The number who were eligible for the study was also lower than expected. The principal reasons for non-eligibility were going into a care home after admission and not having a carer to participate alongside the patient. A future trial could potentially include people going into care homes, but the intervention is currently designed principally around activities in the person’s own home and so redesign would be required. Also, it might not be appropriate to combine the results of participants in their own homes with those in care homes in any future trial. In addition to the lower numbers of eligible patients than expected, some patients were not approached. This could be due to staff capacity, the person being discharged or staff reluctance to approach someone they considered to be very ill or frail.

Despite these challenges, consent rates were promising, and data completeness was generally good at baseline and for those remaining in the trial at follow-up. The successful completion of the intervention among those who remained in the study indicates that, with improved recruitment strategies, a future trial could be feasible. However, retention of participants was lower than expected as many participants withdrew due to death, going into a care home or deterioration in condition. This is not surprising given the frailty often associated with delirium and its poor prognosis.^48^ Indeed, the cognitive and physical barriers inherent to this older population—such as postdelirium fatigue, fluctuating mental capacity, frailty, multimorbidity and the social burden on family members acting as carers—directly contributed to the feasibility challenges encountered. These factors likely explain the high attrition rate between consent and intervention initiation, as the period immediately following hospital discharge is a time of peak vulnerability for older adults with delirium.

This study contributes to a growing body of literature focused on multidisciplinary recovery for survivors of acute illness. Our approach shares theoretical similarities with established frameworks such as the Trauma Medical Home (TMH)^49 50^ and Critical Care Recovery Programme (CCRP),^51 52^ which emphasise proactive, coordinated care to mitigate long-term cognitive and functional decline. However, while the TMH and CCRP models often rely on centralised, clinic-based coordination, RecoverED distinguishes itself by delivering a home-based, therapist-led intervention specifically tailored to the fluctuating cognitive nature of delirium recovery. Positioning the intervention in the home environment was a deliberate strategy to address previously reported barriers in post-ICU and trauma populations, such as the physical and logistical burdens of attending hospital-based clinics.^51^ Despite the 'mixed’ success of prior multidisciplinary models in achieving functional improvements, our study is necessitated by the 'cognitive-functional’ overlap in delirium, where standard geriatric reablement often lacks the specialised psychosocial and cognitive education required to support both the patient and the carer.

Study limitations include the lack of ethnic diversity among participants, as all participants were white, which does not fully represent the broader population of older adults experiencing delirium. This homogeneity limits our understanding of how the intervention may be received across different ethnic backgrounds and cultural contexts. In addition, the reliance on clinical teams to diagnose delirium rather than a standardised research staff-based approach also revealed weaknesses in identification processes, which may have led to missed opportunities for recruitment. There is also potential for selection bias to exist, as not all eligible patients were approached for participation, often due to staff capacity constraints or their perceived frailty. In addition, the home-based delivery model introduces important considerations for generalisability and future implementation. Participation required individuals to live within the geographical reach of the intervention delivery teams and to have access to community rehabilitation services, which may limit applicability to people living in more remote or rural areas or in health systems without comparable post-acute home-based rehabilitation infrastructure. These limitations highlight the need for future studies to adopt more inclusive recruitment strategies, broaden geographic reach and use standardised screening protocols to enhance participation and representation of vulnerable populations in research focused on delirium recovery.

### Conclusions

The development of a comprehensive, multidisciplinary rehabilitation intervention to support recovery from delirium represents a significant step forward in addressing the complex needs of individuals posthospital discharge. The intervention was found to be acceptable to participants and carers, and its delivery by trained RSWs was feasible. However, the study did not meet all thresholds required for progression to a definitive RCT. While the consent rate among eligible participants was strong, intervention completion and retention to 6 month follow-up fell below the ‘green light’ targets. These findings indicate that a definitive RCT would be feasible only if specific modifications are implemented, including enhanced strategies to improve recruitment and geographic reach, mitigate attrition between hospital discharge and intervention initiation, as well as refined methods for longitudinal data collection. Furthermore, future work is required to establish the most appropriate primary outcome measure for this population before proceeding to a large-scale trial.

## Supplementary material

10.1136/bmjopen-2025-102316 online supplemental file 1

## Data Availability

Data are available on reasonable request.
