# Supplementary material for: Rehabilitation intervention to improve Recovery after an Episode of Delirium in adults over 65 years (RecoverED): a multicentre, single-arm feasibility study in NHS acute hospitals in the UK
Source: BMJ Open. 2026 Apr 22;16(4):e102316. doi: 10.1136/bmjopen-2025-102316 (PMC13110589; doi:10.1136/bmjopen-2025-102316)
Supplement: online supplemental file 1 [file bmjopen-16-4-s001.docx]

## Additional File 1:

## The study underwent several substantive amendments that impacted its original design. Notably, the following key changes were made:

- **Midway Intervention Review**: A substantial amendment (dated January 26, 2023) introduced an individual participant midway intervention review, whereby a therapist accompanied the Rehabilitation Support Worker (RSW) during one of the ten intervention sessions. This allowed for real-time evaluation and adjustment of the intervention, ensuring participants received tailored support.
- **Eligibility Criteria Adjustments**: Eligibility criteria were refined (dated October 25, 2023) to specify a "clinical diagnosis of delirium while admitted to the hospital," replacing the previous condition requiring delirium to last for more than 48 hours.
- **Geographical Exclusion**: The eligibility criteria also excluded participants living outside the geographical reach of the intervention teams, focusing the study on those who could realistically receive the intervention.
- **Recruitment Process Modifications**: The design adapted to allow rapid consent and baseline procedures for patients discharged from the hospital within one week, using a face-to-face visit from a clinical researcher, thus streamlining the recruitment process.
- **Compensation for Healthcare Professionals**: A financial compensation of £35 was introduced for healthcare professionals participating in the process evaluation, addressing recruitment challenges (dated February 6, 2024).

The recruitment period for the feasibility study was extended twice to address unforeseen challenges at multiple sites. Delays occurred in opening two of the planned six sites due to difficulties in securing therapists and support workers, alongside slow setup progress. Additionally, two other sites faced issues with poor screening and documentation practices for delirium, compounded by limited staff capacity, resulting in recruitment rates falling below target. Following discussions with the Programme Steering Committee (PSC), sponsor, and funder, the recruitment period was extended first from October 31, 2023, to December 31, 2023, and then to March 31, 2024. However, recruitment at two sites could not continue beyond January 2024 due to persistent staff capacity issues.

All amendments were submitted and approved by the London South East REC, ensuring compliance with ethical standards. The scientific justification for these changes cantered on the need to enhance participant support, improve recruitment, and adapt the study design to logistical realities, thereby strengthening the overall feasibility and impact of the intervention. These modifications were documented and endorsed by the PSC and the funder prior to submission, ensuring alignment with the study's objectives and ethical considerations.

## Additional File 2:

| **Measure** | **Description** | **Time point** |
| --- | --- | --- |
| **Proposed Primary** | | |
| Activities of daily living (ADL) | This will be passed by the Disability Assessment for Dementia (DAD) [38] | Baseline, 6 months |
| **Secondary** | | |
| Activities of daily living (ADL) | As described above for primary outcome | Baseline, 3 months |
| Mobility | This will be assessed using the Timed Up and Go (TUG). | Baseline, 3 months  6 months |
| Delirium persistence or recurrence | This will be assessed by DSM5 criteria [53], with some additional enhancements including use of the Informant Assessment of Geriatric Delirium scale (I-AGeD) [54]. | Baseline, 3 months,  6 months |
| Attention | Assessment of attention using number of months of the year backwards. | Baseline, 3 months,  6 months |
| Level of arousal | Observational Scale of level of Arousal (OSLA) [36] will be used to assess the level of arousal in people with delirium. | Baseline, 3 months,  6 months |
| Cognition | This will be assessed with mini ACE (Mini-ACE) [55] | Baseline, 3 months,  6 months |
| Verbal fluency | Verbal fluency will be assessed using the ‘Animals’ assessment from the mini-ACE assessment. | Baseline, 3 months,  6 months |
| Identity self-continuity | Single item question to assess how the patient participant feels about themselves. | Baseline, 3 months,  6 months |
| Verbal short-term and working memory | This will be assessed with the Digit span test (Forward Digit Span and Reverse Digit Span). | Baseline, 3 months,  6 months |
| Mood assessment | This will be assessed using Geriatric Depression Scale-4 (GDS-4) [56] | 3 months,  6 months |
| Wellbeing | This will be assessed using the ICEpop CAPability measure for Older people (ICECAP-O) [40] | Baseline, 3 months,  6 months |
| Residence category | Residence types | 3 months, 6 months |
| Patient health-related quality of life (HRQL) | This will be assessed using the EQ-5D-5L and EQ-5D-5L proxy [39] | Baseline, 3 months,  6 months |
| Patient HRQL | This will be assessed with the DEMQOL and DEMQOL-Proxy [57] | Baseline, 3 months,  6 months |
| Carer burden | This will be assessed using the Zarit burden interview 12 (ZBI-12) [58] | Baseline, 3 months,  6 months |
| Carer quality of life | This will be assessed using the EQ-5D-5L | Baseline, 3 months,  6 months |
| Carer wellbeing | This will be assessed using the ICEpop CAPability measure for Adults (ICECAP-A) [41] | Baseline, 3 months,  6 months |
| Resource use | Data will be collected via a proxy Resource Use Questionnaire (RUQ). | Baseline, 3 months,  6 months |

## Additional File 3:

The economic evaluation component of this study aimed to assess the feasibility of conducting a full, policy-relevant, cost-effectiveness analysis of the intervention alongside a future RCT.

The resources required to deliver the intervention were identified through discussion with the research team, intervention developers and providers, and included staff time, travel, training, supervision, and materials. These resources were measured within-trial using participant-level case report forms, with additional information provided from intervention developers and providers, and were valued using nationally recognised UK unit costs, including the National Schedule of NHS Costs [59]. Unit Costs for Health and Social Care [60] and the Adult Social Care Activity and Finance Report [61] for 2022/23. Where published sources were not available, costs were identified in consultation with the intervention developers and providers.

To capture other relevant resources used during the study period, a bespoke resource use questionnaire (RUQ) was developed by the research team and the Patient Advisory Group for the study, informed by previous resource use instruments and core items for resource use measures [42, 43]. The RUQ was designed for proxy completion by carers, and included NHS and personal social services (PSS) resources, ie primary, secondary and social care resource use, as well as broader societal resources, including informal care/support, Voluntary, Community and Social Enterprise Sector services and support, and additional participant purchases and costs. The RUQ was administered at baseline and at three and six month follow-up. NHS and PSS resource use was valued using nationally recognised UK unit costs for health and social care services [59, 60]. Informal care was costed using median hourly earnings sourced from the ONS Annual Survey of Hours and Earnings 2023 [62], and costs of additional purchases were reported directly by carers completing the RUQ. Use of Voluntary, Community and Social Enterprise Sector services and support was rarely reported, and these were therefore not costed for the purposes of the feasibility study.

The intended primary economic outcome measure for a definitive trial was quality-adjusted life years (QALYs), derived from health state values obtained using the EQ-5D-5L health-related quality of life (HRQL) instrument [39]. Further information on the HRQL of patient participants was obtained from a dementia-specific measure, the DEMQOL [63]. Due to the potential for some patient participants to lack capacity to complete questionnaires at one or more time-points, both self-report and proxy-report versions of the EQ-5D-5L (EQ-5D-5L Proxy Version 2) and DEMQOL (DEMQOL-Proxy) were administered to all patient participants at all time-points, with the carer providing the proxy reports. In addition, information on patient and carer wellbeing was collected using the relevant version of the ICEpop CAPability (ICECAP) measures, and used to calculate wellbeing-adjusted life-years (WALYs). Data on HRQL and wellbeing for patient and carer participants were collected within the trial at baseline, and at three and six month follow-ups, as set out in Table 7 below.

**Economic outcome measures**

| **Data** | **Instrument(s)** | **Completion** |
| --- | --- | --- |
| Patient participants’ HRQL | EQ-5D-5L  DEMQOL  EQ-5D-5L Proxy Version 2  DEMQOL-Proxy | Self-report by patients  Proxy completion by carers |
| Patient participants’ wellbeing | ICECAP-O | Self-report by patients or proxy completion by carers |
| Carers’ HRQL | EQ-5D-5L | Self-report by carers |
| Carers’ wellbeing | ICECAP-A | Self-report by carers |

Health state values were derived from responses to the EQ-5D-5L and EQ-5D-5L Proxy Version 2, by mapping between EQ-5D-5L responses and the published UK health state value set for the EQ-5D-3L using an approved algorithm [64], as recommended by the National Institute for Health and Care Excellence (NICE) [65]. Health state values were derived from responses to the DEMQOL and the DEMQOL-Proxy, using published tariffs [66]. Wellbeing values were derived from responses to the ICECAP-O and ICECAP-A, using the published sources of values for these instruments [40, 67]. QALYs and WALYs were estimated from the derived health state values and wellbeing values respectively through application of standard area-under-the-curve (AUC) methods, using baseline, Month Three and Month Six assessments.

## Additional File 4:

**Resource use over follow-up period**

| **NHS/PSS resource use (contacts)** | **Obs** | **Used** | **Mean** | **SD** | **Min** | **Max** |
| --- | --- | --- | --- | --- | --- | --- |
| **Hospital-based services** |  |  |  |  |  |  |
| Accident and Emergency visits | 10 | 3 | 0.70 | 1.34 | 0 | 4 |
| Inpatient stays (number of nights) | 10 | 4 | 24.00 | 58.54 | 0 | 188 |
| Day case admissions | 10 | 2 | 0.80 | 1.75 | 0 | 5 |
| OT at hospital | 9 | 2 | 3.56 | 9.94 | 0 | 30 |
| Physiotherapist at hospital | 9 | 2 | 5.67 | 16.63 | 0 | 50 |
| Geriatrician at hospital | 10 | 2 | 2.20 | 6.29 | 0 | 20 |
| Geriatrician by telephone/online | 10 | 1 | 0.20 | 0.63 | 0 | 2 |
| Psychiatrist at hospital | 10 | 1 | 0.10 | 0.32 | 0 | 1 |
| Psychiatrist by telephone/online | 10 | 0 | 0 | 0 | 0 | 0 |
| Neurologist at hospital | 10 | 1 | 0.10 | 0.32 | 0 | 1 |
| Neurologist by telephone/online | 10 | 0 | 0 | 0 | 0 | 0 |
| Paramedic | 9 | 3 | 0.67 | 1.12 | 0 | 3 |
| **Community-based services** |  |  |  |  |  |  |
| GP at health centre | 10 | 5 | 1.10 | 1.66 | 0 | 5 |
| GP by telephone/online | 8 | 4 | 2.13 | 2.85 | 0 | 7 |
| GP at home | 8 | 3 | 1.00 | 1.51 | 0 | 4 |
| Practice nurse at health centre | 10 | 3 | 0.50 | 0.97 | 0 | 3 |
| Practice nurse by telephone/online | 8 | 1 | 0.38 | 1.06 | 0 | 3 |
| Practice nurse at home | 9 | 6 | 4.89 | 6.60 | 0 | 16 |
| Mental health nurse at health centre | 10 | 1 | 0.10 | 0.32 | 0 | 1 |
| Mental health nurse by telephone/online | 10 | 0 | 0 | 0 | 0 | 0 |
| Mental health nurse at home | 10 | 1 | 0.10 | 0.32 | 0 | 1 |
| OT at home | 8 | 7 | 8.50 | 13.28 | 0 | 39 |
| OT by telephone/online | 9 | 1 | 0.11 | 0.33 | 0 | 1 |
| Physiotherapist at health centre | 10 | 0 | 0 | 0 | 0 | 0 |
| Physiotherapist at home | 8 | 2 | 0.75 | 1.75 | 0 | 5 |
| Physiotherapist by telephone/online | 8 | 5 | 3.88 | 4.58 | 0 | 11 |
| Social worker at home | 10 | 3 | 0.60 | 1.07 | 0 | 3 |
| Social worker by telephone/online | 10 | 4 | 1.00 | 1.70 | 0 | 5 |
| Direct Payment (amount received) | 10 | 0 | 0 | 0 | 0 | 0 |
| **Other support services** |  |  |  |  |  |  |
| Rehabilitation unit (number of days) | 10 | 1 | 10.50 | 33.20 | 0 | 105 |
| Respite care unit (number of days) | 10 | 2 | 2.70 | 6.70 | 0 | 21 |
| Day care centre (number of days) | 9 | 0 | 0 | 0 | 0 | 0 |
| Home care | 10 | 6 | 70.90 | 104.24 | 0 | 336 |
| Extra help at home | 9 | 0 | 0 | 0 | 0 | 0 |
| Groups for people with dementia | 10 | 0 | 0 | 0 | 0 | 0 |
| Peer-led groups | 10 | 0 | 0 | 0 | 0 | 0 |
| Groups for older people | 10 | 0 | 0 | 0 | 0 | 0 |
| Telephone/online support | 10 | 1 | 0.20 | 0.63 | 0 | 2 |

**Resource use over follow-up period continued**

| **Societal resource use (contacts)** | **Obs** | **Used** | **Mean** | **SD** | **Min** | **Max** |
| --- | --- | --- | --- | --- | --- | --- |
| **Support services** |  |  |  |  |  |  |
| Rehabilitation unit (number of days) | 10 | 0 | 0 | 0 | 0 | 0 |
| Respite care unit (number of days) | 10 | 0 | 0 | 0 | 0 | 0 |
| Day care centre (number of days) | 9 | 0 | 0 | 0 | 0 | 0 |
| Home care | 10 | 0 | 0 | 0 | 0 | 0 |
| Extra help at home | 9 | 0 | 0 | 0 | 0 | 0 |
| Groups for people with dementia | 10 | 1 | 2 | 6.32 | 0 | 20 |
| Peer-led groups | 10 | 0 | 0 | 0 | 0 | 0 |
| Groups for older people | 10 | 0 | 0 | 0 | 0 | 0 |
| Telephone/online support | 10 | 1 | 1.20 | 3.79 | 0 | 12 |
| **Unpaid care** |  |  |  |  |  |  |
| Unpaid care (hours per month) | 9 | 8 | 731.89 | 887.28 | 0 | 2888 |
| Number of days off work for carer | 10 | 1 | 0.90 | 2.85 | 0 | 9 |

| **Own expenses (cost in GBP)** | **Obs** | **Used** | **Mean** | **SD** | **Min** | **Max** |
| --- | --- | --- | --- | --- | --- | --- |
| **Support services** |  |  |  |  |  |  |
| Rehabilitation unit (number of days) | 10 | 0 | 0 | 0 | 0 | 0 |
| Respite care unit (number of days) | 10 | 0 | 0 | 0 | 0 | 0 |
| Day care centre (number of days) | 9 | 0 | 0 | 0 | 0 | 0 |
| Home care | 10 | 2 | 708.25 | 2011.35 | 0 | 6400 |
| Extra help at home | 9 | 3 | 140.89 | 213.34 | 0 | 490 |
| Groups for people with dementia | 10 | 0 | 0 | 0 | 0 | 0 |
| Peer-led groups | 10 | 0 | 0 | 0 | 0 | 0 |
| Groups for older people | 10 | 0 | 0 | 0 | 0 | 0 |
| Telephone/online support | 10 | 0 | 0 | 0 | 0 | 0 |
| **Other expenses** |  |  |  |  |  |  |
| Over the counter medication | 7 | 2 | 38.57 | 66.44 | 0 | 150 |
| Complementary therapies | 6 | 1 | 80.00 | 195.96 | 0 | 480 |

**Key:**

Obs: total number of valid observations. This indicates the amount of missing data per item.

Used: total number of people who reported using the service and provided contacts/cost data.

**Resource use over follow-up period costed**

| **NHS/PSS resource use** | **Obs** | **Mean** | **SD** | **Min** | **Max** |
| --- | --- | --- | --- | --- | --- |
| **Hospital-based services** |  |  |  |  |  |
| Accident and Emergency visits | 10 | 187.73 | 358.69 | 0 | 1072.72 |
| Inpatient stays (number of nights) | 10 | 2478.76 | 2133.38 | 0 | 4131.27 |
| Day case admissions | 10 | 490.70 | 1074.13 | 0 | 3066.85 |
| OT at hospital | 9 | 421.33 | 1177.74 | 0 | 3555.00 |
| Physiotherapist at hospital | 9 | 575.90 | 1689.93 | 0 | 5081.50 |
| Geriatrician at hospital | 10 | 750.64 | 2144.71 | 0 | 6824.00 |
| Geriatrician by telephone/online | 10 | 52.50 | 166.01 | 0 | 524.98 |
| Psychiatrist at hospital | 10 | 25.59 | 80.92 | 0 | 255.90 |
| Neurologist at hospital | 10 | 22.30 | 70.52 | 0 | 222.99 |
| Paramedic | 9 | 270.57 | 453.77 | 0 | 1217.58 |
| **Community-based services** |  |  |  |  |  |
| GP at health centre | 10 | 53.90 | 81.50 | 0 | 245.00 |
| GP by telephone/online | 8 | 56.04 | 75.17 | 0 | 184.59 |
| GP at home | 8 | 91.96 | 139.03 | 0 | 367.84 |
| Practice nurse at health centre | 10 | 6.85 | 13.30 | 0 | 41.07 |
| Practice nurse by telephone/online | 8 | 3.39 | 9.59 | 0 | 27.12 |
| Practice nurse at home | 9 | 159.77 | 215.81 | 0 | 522.88 |
| Mental health nurse at health centre | 10 | 24.93 | 78.85 | 0 | 249.34 |
| Mental health nurse at home | 10 | 24.93 | 78.85 | 0 | 249.34 |
| OT at home | 8 | 1055.19 | 1648.24 | 0 | 4841.46 |
| OT by telephone/online | 9 | 0.64 | 1.93 | 0 | 5.79 |
| Physiotherapist at home | 8 | 299.93 | 354.54 | 0 | 851.40 |
| Physiotherapist by telephone/online | 8 | 4.34 | 10.15 | 0 | 28.95 |
| Social worker at home | 10 | 38.16 | 68.37 | 0 | 190.80 |
| Social worker by telephone/online | 10 | 5.79 | 9.84 | 0 | 28.95 |
| Rehabilitation unit | 10 | 413.13 | 1306.42 | 0 | 4131.27 |
| Respite care facility/unit | 10 | 324.62 | 805.63 | 0 | 2524.83 |
| Home care | 10 | 729.21 | 1072.13 | 0 | 3455.76 |
| **TOTAL NHS/PSS COSTS** | **3** | **4496.36** | **312.79** | **4163.95** | **4784.89** |
| **Societal costs** |  |  |  |  |  |
| **Own expenses** |  |  |  |  |  |
| Home care | 10 | 708.25 | 2011.35 | 0 | 6400.00 |
| Extra help at home | 9 | 140.89 | 213.34 | 0 | 490.00 |
| Over the counter medication | 7 | 38.57 | 66.44 | 0 | 150.00 |
| Complementary therapies | 6 | 80.00 | 195.96 | 0 | 480.00 |
| **Unpaid care** |  |  |  |  |  |
| Unpaid care | 9 | 34757.40 | 42136.84 | 0 | 137151.10 |
| Days off work taken by carer | 10 | 99.73 | 315.37 | 0 | 997.29 |
| **TOTAL SOCIETAL COSTS** | **5** | **40710.04** | **55484.04** | **1329.72** | **137151.10** |
| **TOTAL NHS/PSS AND SOCIETAL COSTS** | **2** | **18862.08** | **18027.64** | **6114.61** | **31609.54** |
