# Supplementary material for: Rehabilitation intervention to improve Recovery after an Episode of Delirium in adults over 65 years (RecoverED): a multicentre, single-arm feasibility study in NHS acute hospitals in the UK
Source: BMJ Open. 2026 Apr 22;16(4):e102316. doi: 10.1136/bmjopen-2025-102316 (PMC13110589; doi:10.1136/bmjopen-2025-102316)
Supplement: Supplementary file 2 [file bmjopen-2025-102316.reviewer_comments.pdf]

PEER REVIEW HISTORY

BMJ Open publishes all reviews undertaken for accepted manuscripts. Reviewers are asked to complete a checklist review form and are provided with free text boxes to elaborate on their assessment. These free text comments are reproduced below.

ARTICLE DETAILS

Title (Provisional)

A rehabilitation intervention to improve Recovery after an Episode of Delirium in adults over 65 years (RecoverED): a multi-centre, single-arm feasibility study in NHS acute hospitals in the United Kingdom

Authors

Allan, Louise; Um, Jinpil; O'Connell, Abby; Raghuraman, Shruti; Bingham, Alison; Laberick, Abigail; Chandler, Kirstie; Connors, James; Mahmoud, Aseel; Hawton, Annie; Goodwin, Elizabeth; Morgan-Trimmer, Sarah; Goodwin, Victoria; Ukoumunne, Obioha C; Jackson, Thomas; Richardson, Sarah J; Collier, Lesley; Glasby, Jon; Whale, Bethany; Dawe, Phoebe; Burnett-Fry, Naomi; Clare, Linda

---

VERSION 1 - REVIEW

**Reviewer**	**1**
**Name**	**Schimböck, Florian**
**Affiliation**	**Kiel University**
**Date**	**29-Aug-2025**
**COI**	**None**

Thank you for the opportunity to review the manuscript “A rehabilitation intervention to improve Recovery after an Episode of Delirium in adults over 65 years (RecoverED): a multi-centre, single arm feasibility study”.

The manuscript presents an engaging and relevant study that makes a valuable contribution to the field of delirium research and care. The article is clearly structured and well written, which makes it accessible and enjoyable to read. Overall, only minor revisions are needed to further strengthen the clarity and impact of the work. I hope the authors find my suggestions helpful.

General: In the manuscript different terms like “RecoverED study”, “RecoverED programme” and “RecoverED intervention” are used. While minor, ensuring consistent use of “study” and “intervention” when referring to the current research being reported can improve precision.

Abstract (p. 2, 5-7): “To test a new, theory-informed rehabilitation intervention …”. There is no theoretical basis or framework described or cited in the introduction or the intervention

which explains the theoretical underpinnings of the intervention as stated in the abstract. Provide a brief summary of the key theoretical underpinnings to justify the "theory-informed" claim in the abstract. This would help readers understand the conceptual basis of the intervention without needing to consult another paper immediately.

Introduction (p. 4, 44-47): "Despite growing recognition of the significance of delirium, there remains a notable gap in research focusing on the post-hospitalisation phase and the support needs of affected individuals and their caregivers." While this statement identifies a gap, it could be more impactful by briefly explaining why this gap is particularly problematic or under-addressed. For instance, is it because most research focuses on acute management, or because post-discharge care is complex and fragmented?

Aims (p. 4, 54 – p. 5, 3-8): The objectives are listed clearly, but the phrasing "Specifically, it aimed to assess the feasibility of the rehabilitation intervention ... and evaluate its acceptability ... Additionally, the study aimed to explore the feasibility of delivering the intervention ... test the feasibility of collecting data ... and iteratively refine the intervention ..." feel a bit repetitive with "aimed to" and "feasibility". Suggestion: Rephrase to vary sentence structure and emphasize the distinct aspects of feasibility being assessed.

Design (p. 5, 17-18): "… a mixed-methods design combining quantitative and qualitative approaches …". While stated, the qualitative methods are only briefly mentioned (p. 8, 28-29: "Qualitative interviews were conducted …"). The design section could benefit from a slightly more explicit statement of how the mixed methods were integrated (e.g., sequential, concurrent, explanatory, exploratory) and why this approach was chosen for a feasibility study.

Setting (p. 5, 33-34): While the cities are listed, a brief description of the type of hospitals (e.g., tertiary care, district general hospitals, university hospitals) could provide useful context regarding the patient population and healthcare environment.

Eligibility (p. 5, 43-45): "… diagnosed with delirium during their hospital stay …". Given the discussion in the "discussion" section about weaknesses in screening processes, it would be beneficial to briefly mention how this diagnosis was typically made (e. g., clinical judgment, positive diagnostic tools used by the clinical team, ICD/DSM diagnosis by a physician) in the eligibility criteria, even if the research team later confirmed it. This sets the stage for the later discussion of recruitment challenges.

Recruitment (p. 6, 5-8): It would be useful to specify who performed this assessment (e. g., nursing staff, medical team) and if there was any variability in the routine application of these tools across the six sites. This directly relates to the later discussion about "weaknesses in screening processes" (p. 15, 41-44).

Intervention (p. 6, 19-43): The "RecoverED" intervention is well-described; however, there are no theoretical underpinnings described and/or cited as the abstract implicates.

Intervention (p. 6, 44-50): Briefly mention the content of the training beyond just “flexibility and accessibility” and “bite-sized modules”. What were the key skills or knowledge areas covered? This adds to replicability.

Feasibility and acceptability (p. 6, table 1): This table is excellent for clearly outlining objectives and outcomes. Consider adding a column for who collected the data for each outcome measure (e. g., research nurse, therapist, participant/carer). For “The acceptability of the intervention assessed during the process evaluation”, briefly state how acceptability was measured (e. g., qualitative interviews, questionnaires). This is partially covered in the process evaluation section, but a cross-reference or brief mention here would be helpful.

Process evaluation (p. 8, 19-22): As mentioned previously, briefly describing the core components of this logic model or citing the “previous research” would strengthen this section.

Process evaluation (p. 8, 26-32): Briefly state the purpose of these interviews beyond “insights into the acceptability ... implementation barriers, and facilitators.” For example, were they semi-structured? How many interviews were aimed for? I know there will be a separate paper, but only a few more details.

Assessments (p. 8, 44-45): “detailed delirium assessment was completed using standardised Diagnostic and Statistical Manual of Mental Disorders (DSM)-5 criteria”. Clarify who performed this assessment (e. g., research nurse, clinician) and if it was a retrospective review or a direct assessment. This is important given the earlier discussion about clinical diagnosis vs. research screening.

Results (p. 10, figure 1): The footnote clarifies “Some participants ineligible for more than one reason”. It would be helpful to briefly state the primary reasons for ineligibility here, as it's a major finding. This is discussed in the “discussion”, but a brief preview here would be nice.

Data completeness and clinical outcomes (p. 12, 6-9): “Six-month follow-ups were completed within the +/- 2 week window for 53% of all participants, and for all 10 of those who were expected to provide data at that wave.”. Maybe rephrase for more clarity: “Six-month follow-ups were completed within the +/- 2 week window for all 10 participants who remained in the study until the final follow-up (53% of the initial 19 participants).”

Intervention fidelity and acceptability (p. 14, 54-56): “Detailed findings related to intervention fidelity and acceptability will be reported in a separate paper.”. While a separate paper is planned, a very brief summary of the key findings would strengthen the current paper's conclusions regarding feasibility and readiness for an RCT. Without this, the reader has to take the “acceptability” conclusion on faith.

Discussion (p. 15, 19-21): “Adherence to the intervention was satisfactory, with 53% of the participants attending six or more sessions.”. While stated as “satisfactory” it's worth noting

that this 53% is below the “green light” progression criterion of “>70% participants attend >60% of sessions” from Table 2. This discrepancy should be explicitly addressed.

Crucial missing element: The discussion mentions the progression criteria in the methods and the results provide the numbers, but the discussion does not explicitly state whether the study met its own progression criteria for a definitive RCT. Suggestion: Add a dedicated paragraph or integrate into the existing discussion a clear statement on whether each “green light” criterion from Table 2 was met.

Conclusions (p. 16, 38-52): Nuancing the conclusion based on the progression criteria. If the criteria for progression were not fully met, the discussion should clearly state what further work is needed before a full RCT.

**Reviewer**	**2**
**Name**	**Fuchita, Mikita**
**Affiliation**	**University of Colorado**
**Date**	**11-Dec-2025**
**COI**	**None**

Summary

This feasibility study enrolled 19 older adults (>65 years) with delirium who were discharged from acute care settings and followed them longitudinally for 6 months to deliver up to 10 home-based multidisciplinary rehabilitation sessions. The study reports a 9% eligibility rate, 53% consent rate, 53% intervention completion rate (≥60% of sessions), and 53% retention at 6 months. Overall, the study is rigorously conducted and well-written. The authors clearly summarize their findings, and the inclusion of a process evaluation and economic analysis adds depth and value. My major feedback concerns the conclusion to proceed to a definitive trial. Based on the feasibility challenges described, it is not yet convincing that a larger trial is justified unless the investigators can demonstrate that the identified barriers are modifiable and provide a clear plan to address them. Importantly, the feasibility study has inherent value even if the ultimate conclusion is that a full-scale trial is not feasible. This work addresses an important gap in delirium care, and the study’s scientific contribution is not diminished by a decision not to proceed.

Major Comments

1. Determination of Feasibility

Among the feasibility outcomes, only the consent rate clearly met the “green light” criterion. Intervention adherence and retention were suboptimal and fell within intermediate ranges. Based on these data alone, one could reasonably conclude that feasibility was not demonstrated. The authors should contextualize their results by comparing them with

feasibility benchmarks from other longitudinal delirium or post-acute geriatric rehabilitation studies. It would be helpful to discuss whether lower intervention fidelity and retention are inherent to this population, potentially modifiable through protocol redesign, or likely to remain a limiting factor for a future trial. If the authors believe that a larger trial is still justified, they should provide supporting rationale, including: (a) which feasibility concerns are modifiable; (b) specific strategies to address them; and (c) an explanation of how these changes would plausibly improve recruitment, adherence, and retention.

At present, the recommendation to move forward appears driven more by optimism than by the data presented. The challenges documented in this single-arm study—where all participants received an intervention with possible benefit—suggest that recruitment and retention in a future RCT, particularly in the control arm, may be even more difficult. Additionally, it is unclear how the pre-defined progression criteria for determining feasibility were derived. A 60% retention target may be a low threshold and risks substantial selection bias; the rationale should be discussed. Providing the rationale and citing benchmarks from similar longitudinal studies of older adults after acute hospitalization would greatly strengthen this interpretation.

2. Contextualizing the Intervention Within Prior Literature

The manuscript would benefit from a more robust discussion of prior longitudinal multidisciplinary interventions aimed at improving long-term recovery after acute illness, such as the Trauma Medical Home model (doi:10.1001/jamasurg.2024.1043) and the Critical Care Recovery Clinic (doi:10.1001/jamanetworkopen.2023.53158). Positioning the current work within this context would help readers understand: how this model compares with prior efforts, how this study aimed to address previously reported barriers (e.g., retention, intervention fidelity), and why this particular approach may be necessary despite prior mixed or negative findings.

3. Missing Discussion of the Unique Challenges Among Older Adults

Although the study focuses exclusively on older adults with delirium, the manuscript does not articulate why this population was targeted. A brief discussion of the disproportionate burden of delirium on older adults—and the associated cognitive, physical, and social barriers to longitudinal study participation—would strengthen the rationale for the study design. It would also contextualize the feasibility challenges the authors encountered.

4. Proposed Primary Outcome

The primary clinical outcome (DAD) is introduced only in the Results section. It should be described in the Methods with justification for its selection. The authors should also clarify whether the DAD is validated in populations without dementia, as most participants in this study did not have a dementia diagnosis. This is important for interpreting both feasibility and potential outcome selection for a future trial.

5. Generalizability of Home-Based Intervention Delivery

The home-based delivery model may limit generalizability to individuals living farther from the clinical site or in regions without comparable home rehabilitation services. This was described as an enrollment barrier but is also a broader implementation limitation that warrants discussion.

Minor Comments

1. Eligibility and Screening

The manuscript describes how patients were assessed for eligibility but does not describe the screening procedures used to identify potential participants prior to eligibility assessment. A brief description would improve transparency and reproducibility.

2. Acronyms and Terminology

Spell out NHS at first use for international readers. Consider revising the acronym "RecoverED," as "ED" may be misinterpreted as Emergency Department, despite not being the study setting.

3. Personnel Roles

Please define the role and training of the "rehabilitation support worker." It is unclear whether this role is distinct from the "healthcare support worker" referenced elsewhere. Consistent terminology would improve clarity.

4. Assessment Procedures

Baseline assessments were performed either during hospitalization or after discharge, whereas the abstract only mentions assessments after discharge. Please clarify for consistency. In addition, listing the key assessment instruments used (even briefly) would help readers understand feasibility and study burden.

5. Sample Size Considerations

I encourage the authors not to include a sample size calculation as part of the feasibility objective, since preliminary efficacy signals from such a small sample are not meaningful. The initial target enrollment was 60 participants; however, only 19 were enrolled. Please comment on whether this discrepancy itself informs feasibility conclusions.

6. Feasibility Outcome Tables

Tables 1 and 2 include overlapping information, which may confuse readers. Consider merging or restructuring them. As noted above, please state the rationale for the progression criteria and whether thresholds were defined a priori.

7. CONSORT Flow Diagram

Please include detailed reasons for ineligibility, withdrawal, and loss to follow-up.

8. Citation

Reference #30 appears incorrect. Please also cite foundational work that informed the logic model described in the Methods (process evaluation section).

9. Interpretation of Clinical Outcomes

The Discussion suggests that participation in the intervention was associated with improvements in DAD scores over 6 months. This change may reflect natural recovery after hospitalization rather than an intervention effect. The authors should avoid causal language and, if possible, cite expected recovery trajectories in similar populations.

10. Conclusion

The conclusion links high consent rate and participant engagement to potential clinical benefit; these metrics reflect feasibility, not effectiveness, and should not be interpreted as indicators of intervention impact.

VERSION 1 - AUTHOR RESPONSE

	**Reviewer 1**	
	Specific Comments	***Responses***
1	General: In the manuscript different terms like "RecoverED study", "RecoverED programme" and "RecoverED intervention" are used. While minor, ensuring consistent use of "study" and "intervention" when referring to the current research being reported can improve precision.	Thank you for noting this. We have revised the manuscript to ensure consistent terminology throughout.
2	Abstract (p. 2, 5-7): "To test a new, theory-informed rehabilitation intervention …". There is no theoretical basis or framework described or cited in the introduction or the intervention which explains the theoretical underpinnings of the intervention as stated in the abstract. Provide a brief summary of the key theoretical underpinnings to justify the "theory-informed" claim in the abstract. This would help readers understand the conceptual basis of the intervention without needing to consult another paper immediately.	The intervention was developed based on a realist-informed programme theory, which was completed within the RecoverED project. This work has just been accepted for publication. We have cited this in the introduction where we introduce the RecoverED project.
3	Introduction (p. 4, 44-47): "Despite growing recognition of the significance of delirium, there remains a notable gap in research focusing on the post-hospitalisation phase and the support needs of affected individuals and their caregivers." While this statement identifies a gap, it could be more impactful by briefly explaining why this gap is particularly problematic or under-addressed. For instance, is it because most research focuses on acute management, or because post-discharge care is complex and fragmented?	We agree that the clinical implications of this research gap needed further emphasis. We have added text to the Introduction clarifying that the focus on acute management has left post-discharge care fragmented, which places an undue burden on patients and caregivers during a period of significant vulnerability.

4	Aims (p. 4, 54 – p. 5, 3-8): The objectives are listed clearly, but the phrasing "Specifically, it aimed to assess the feasibility of the rehabilitation intervention ... and evaluate its acceptability ... Additionally, the study aimed to explore the feasibility of delivering the intervention ... test the feasibility of collecting data ... and iteratively refine the intervention ..." feel a bit repetitive with "aimed to" and "feasibility". Suggestion: Rephrase to vary sentence structure and emphasize the distinct aspects of feasibility being assessed.	We have revised the Aims section to improve readability and reduce the repetitive use of 'aimed to' and 'feasibility.' The new phrasing more clearly distinguishes between the primary evaluation of acceptability and the secondary objectives related to process evaluation and outcome measure validation.
5	Design (p. 5, 17-18): "… a mixed-methods design combining quantitative and qualitative approaches …". While stated, the qualitative methods are only briefly mentioned (p. 8, 28-29: "Qualitative interviews were conducted …"). The design section could benefit from a slightly more explicit statement of how the mixed methods were integrated (e.g., sequential, concurrent, explanatory, exploratory) and why this approach was chosen for a feasibility study.	Thank you for this point. We have revised the 'Design' section. We have added a sentence explaining that this approach was selected to provide explanatory power; while quantitative data established feasibility metrics (e.g., recruitment rates), the qualitative data offered necessary insight into the 'how' and 'why' behind these results to inform intervention refinement.
6	Setting (p. 5, 33-34): While the cities are listed, a brief description of the type of hospitals (e.g., tertiary care, district general hospitals, university hospitals) could provide useful context regarding the patient population and healthcare environment.	We agree that this context is valuable. We have revised the 'Setting' section to specify that all recruitment sites were university teaching hospitals providing tertiary care. We have also added detail regarding the healthcare environment, noting that some sites delivered the intervention through the acute hospital's own services while others partnered with community Trusts, reflecting the diverse organisational pathways typical of the UK healthcare landscape.
7	Eligibility (p. 5, 43-45): "… diagnosed with delirium during their hospital stay …". Given the discussion in the "discussion" section about weaknesses in screening processes, it would be beneficial to briefly mention how this diagnosis was typically made (e. g., clinical judgment, positive diagnostic tools used by the clinical team, ICD/DSM diagnosis by a physician) in the eligibility criteria, even if the research team later confirmed it. This sets the stage for the later discussion of recruitment challenges.	We have revised the eligibility section to clarify how delirium was typically identified. Specifically, we now state that participants were identified through routine clinical care, usually following a positive delirium screen and confirmation of a clinical diagnosis by the treating clinical team.
8	Recruitment (p. 6, 5-8): It would be useful to specify who performed this assessment (e. g., nursing staff, medical team) and if there was any variability in the routine application of these tools across the six sites. This directly relates to the later discussion about "weaknesses in screening processes" (p. 15, 41-44).	Thank you for this helpful comment. We have revised the section to clarify who undertook delirium screening and how this was implemented across sites. The manuscript now specifies that delirium screening was performed by members of the clinical care team (including nursing and medical staff) as part of routine ward practice, with patients who screened positive subsequently discussed with a clinical researcher embedded within the clinical team to confirm whether a clinical diagnosis of delirium had been made. We have also stated that the consistency of screening

		reflected usual care and varied across the six participating sites.
9	Intervention (p. 6, 19-43): The “RecoverED” intervention is well-described; however, there are no theoretical underpinnings described and/or cited as the abstract implicates.	We have revised this section to describe the theoretical basis of the RecoverED intervention. The manuscript now states that RecoverED was developed using a realist-informed approach and the Medical Research Council framework for complex interventions, underpinned by an explicit programme theory and logic model identifying physical, cognitive, and psychosocial mechanisms of recovery. We clarify that the published development paper (Bingham et al., 2025) describes how this programme theory was operationalised into a multicomponent complex intervention, while the theoretical foundations and development of the programme theory itself are detailed in the PLOS ONE programme theory paper (Raghuraman et al., 2026), which we now cite accordingly.
10	Intervention (p. 6, 44-50): Briefly mention the content of the training beyond just “flexibility and accessibility” and “bite-sized modules”. What were the key skills or knowledge areas covered? This adds to replicability.	We have expanded the description of the training programme to briefly specify its core content. The revised text now outlines the key knowledge and skill areas covered. This additional detail improves transparency and supports replicability while maintaining a concise description.
11	Feasibility and acceptability (p. 6, table 1): This table is excellent for clearly outlining objectives and outcomes. Consider adding a column for who collected the data for each outcome measure (e. g., research nurse, therapist, participant/carer). For “The acceptability of the intervention assessed during the process evaluation”, briefly state how acceptability was measured (e. g., qualitative interviews, questionnaires). This is partially covered in the process evaluation section, but a cross-reference or brief mention here would be helpful.	A column specifying who collected the data for each outcome measure has been added to Table 1, as suggested. In addition, for outcomes relating to intervention acceptability, we have clarified that acceptability was assessed through the embedded process evaluation using semi-structured qualitative interviews with participants, carers and intervention staff.
12	Process evaluation (p. 8, 19-22): As mentioned previously, briefly describing the core components of this logic model or citing the “previous research” would strengthen this section.	We have now cited the recently accepted publication which details the development of the programme theory underlying the intervention, as well as the paper which details the development of the intervention using the programme theory as the foundation.
13	Process evaluation (p. 8, 26-32): Briefly state the purpose of these interviews beyond “insights into the acceptability ... implementation barriers, and facilitators.” For example, were they semi-structured? How many interviews were aimed for? I know there will be a separate paper, but only a few more details.	We have updated the revised manuscript to expand on the purpose, data sources, and analytic methods underpinning the process evaluation, clarifying how multiple qualitative and quantitative data sources were used to examine implementation fidelity and acceptability. This can be found under Methods -- Design -- Process evaluation.

14	Assessments (p. 8, 44-45): “detailed delirium assessment was completed using standardised Diagnostic and Statistical Manual of Mental Disorders (DSM)-5 criteria”. Clarify who performed this assessment (e. g., research nurse, clinician) and if it was a retrospective review or a direct assessment. This is important given the earlier discussion about clinical diagnosis vs. research screening.	We have revised the Assessments section to clarify that the DSM-5 delirium assessment was undertaken by trained research nurses or equivalent as a research confirmation of delirium.
15	Results (p. 10, figure 1): The footnote clarifies “Some participants ineligible for more than one reason”. It would be helpful to briefly state the primary reasons for ineligibility here, as it's a major finding. This is discussed in the “discussion”, but a brief preview here would be nice.	We have revised the footnote to Figure 1 to briefly indicate the primary reasons for ineligibility.
16	Data completeness and clinical outcomes (p. 12, 6-9): “Six-month follow-ups were completed within the +/- 2 week window for 53% of all participants, and for all 10 of those who were expected to provide data at that wave.”. Maybe rephrase for more clarity: “Six-month follow-ups were completed within the +/- 2 week window for all 10 participants who remained in the study until the final follow-up (53% of the initial 19 participants).”	We have rephrased the sentence to improve clarity, as suggested.
17	Intervention fidelity and acceptability (p. 14, 54-56): “Detailed findings related to intervention fidelity and acceptability will be reported in a separate paper.”. While a separate paper is planned, a very brief summary of the key findings would strengthen the current paper's conclusions regarding feasibility and readiness for an RCT. Without this, the reader has to take the “acceptability” conclusion on faith.	We agree that a brief summary of key findings would strengthen the paper. We have now added a short summary of the main process evaluation findings in the results section.
18	Discussion (p. 15, 19-21): “Adherence to the intervention was satisfactory, with 53% of the participants attending six or more sessions.”. While stated as “satisfactory” it's worth noting that this 53% is below the “green light” progression criterion of “>70% participants attend >60% of sessions” from Table 2. This discrepancy should be explicitly addressed.	We have revised the Discussion to acknowledge that although 53% of participants attended six or more intervention sessions, this level of adherence fell below the pre-specified “green light” progression criterion of ≥70% of participants attending ≥60% of sessions, as outlined in Table 2. The revised text now describes adherence as moderate rather than satisfactory.
19	Crucial missing element: The discussion mentions the progression criteria in the methods and the results provide the numbers, but the discussion does not explicitly state whether the study met its own progression criteria for a definitive RCT. Suggestion: Add a dedicated paragraph or integrate into the existing discussion a clear statement on whether each “green light” criterion from Table 2 was met.	We have revised text now clearly states which criteria were met and which were not.

20	Conclusions (p. 16, 38-52): Nuancing the conclusion based on the progression criteria. If the criteria for progression were not fully met, the discussion should clearly state what further work is needed before a full RCT.	Thank you for this important point. We have revised the Conclusion to provide a more transparent and nuanced summary based on our pre-specified progression criteria.

	**Reviewer 2**	
	Specific Comments	***Responses***
1	Determination of Feasibility<br>Among the feasibility outcomes, only the consent rate clearly met the "green light" criterion. Intervention adherence and retention were suboptimal and fell within intermediate ranges. Based on these data alone, one could reasonably conclude that feasibility was not demonstrated. The authors should contextualize their results by comparing them with feasibility benchmarks from other longitudinal delirium or post-acute geriatric rehabilitation studies. It would be helpful to discuss whether lower intervention fidelity and retention are inherent to this population, potentially modifiable through protocol redesign, or likely to remain a limiting factor for a future trial. If the authors believe that a larger trial is still justified, they should provide supporting rationale, including: (a) which feasibility concerns are modifiable; (b) specific strategies to address them; and (c) an explanation of how these changes would plausibly improve recruitment, adherence, and retention.<br><br>At present, the recommendation to move forward appears driven more by optimism than by the data presented. The challenges documented in this single-arm study—where all participants received an intervention with possible benefit—suggest that recruitment and retention in a future RCT, particularly in the control arm, may be even more difficult. Additionally, it is unclear how the pre-defined progression criteria for determining feasibility were derived. A 60% retention target may be a low threshold and risks substantial selection bias; the rationale should be discussed. Providing the rationale and citing benchmarks from similar longitudinal studies of older adults after acute hospitalization would greatly strengthen this interpretation.	We have revised the Discussion and Conclusions to explicitly state that progression criteria were not fully met and that a definitive RCT would only be feasible with substantial protocol modifications. We now contextualise these findings by highlighting the frailty, comorbidity, and acuity of this population, which are likely to constrain intervention fidelity and retention, and note that these challenges may be only partially modifiable. We also outline specific strategies to improve feasibility (enhanced screening, broader eligibility criteria, and retention support strategies) and clarify that optimism regarding a future trial is conditional on these changes.
2	Contextualizing the Intervention Within Prior Literature<br>The manuscript would benefit from a more robust discussion of prior longitudinal multidisciplinary interventions aimed at improving long-term recovery after acute illness, such as the Trauma Medical Home model (doi:10.1001/jamasurg.2024.1043) and the Critical	Thank you for suggesting these important parallels in recovery literature. We have added a paragraph to the Discussion (p. 18) contextualising our study alongside the Trauma Medical Home and Critical Care Recovery Programme frameworks. We have highlighted that while our intervention shares the multidisciplinary goals of these programmes,

	Care Recovery Clinic (doi:10.1001/jamanetworkopen.2023.53158). Positioning the current work within this context would help readers understand: how this model compares with prior efforts, how this study aimed to address previously reported barriers (e.g., retention, intervention fidelity), and why this particular approach may be necessary despite prior mixed or negative findings.	the decision to deliver the intervention in the participant's home was a specific strategic choice to overcome the logistical and physical attendance barriers frequently cited as limitations in clinic-based recovery models.
3	Missing Discussion of the Unique Challenges Among Older Adults<br>Although the study focuses exclusively on older adults with delirium, the manuscript does not articulate why this population was targeted. A brief discussion of the disproportionate burden of delirium on older adults—and the associated cognitive, physical, and social barriers to longitudinal study participation—would strengthen the rationale for the study design. It would also contextualize the feasibility challenges the authors encountered.	Thank you for this suggestion. we have expanded the discussion to link the feasibility results to the cognitive, physical, and social barriers unique to this population (e.g., post-delirium fatigue, fluctuating mental capacity, and carer burden), providing a clearer context for the attrition rates observed.
4	Proposed Primary Outcome<br>The primary clinical outcome (DAD) is introduced only in the Results section. It should be described in the Methods with justification for its selection. The authors should also clarify whether the DAD is validated in populations without dementia, as most participants in this study did not have a dementia diagnosis. This is important for interpreting both feasibility and potential outcome selection for a future trial.	Thank you for this point. We have added the outcome measures and description of the DAD to the Methods section. We have provided a clear justification: we chose the DAD because we anticipate that delirium survivors without dementia still exhibit cognitive deficits, and this scale is more sensitive to "executive" functional changes than standard ADL tools.
5	Generalizability of Home-Based Intervention Delivery: The home-based delivery model may limit generalizability to individuals living farther from the clinical site or in regions without comparable home rehabilitation services. This was described as an enrollment barrier but is also a broader implementation limitation that warrants discussion.	Thank you for highlighting this implementation barrier. We have revised the limitations section of the Discussion to acknowledge home-based delivery as a broader implementation limitation, beyond its role as an enrollment barrier.
6	Eligibility and Screening:<br>The manuscript describes how patients were assessed for eligibility but does not describe the screening procedures used to identify potential participants prior to eligibility assessment. A brief description would improve transparency and reproducibility.	Thank you for this helpful suggestion and agree that clearer description of the screening procedures strengthens transparency and reproducibility. In response, we have revised the Eligibility and Recruitment section to describe how potential participants were identified prior to formal eligibility assessment.
7	Acronyms and Terminology:<br>Spell out NHS at first use for international readers. Consider revising the acronym “RecoverED,” as “ED” may be misinterpreted as Emergency Department, despite not being the study setting.	We have spelled out “National Health Service (NHS)” at first use to aid international readers. We have also clarified the meaning of the study acronym RecoverED at first mention, explicitly stating that “RecoverED” refers to “recovery after an Episode of Delirium” and not to an

		emergency department setting, to avoid potential confusion.
8	Personnel Roles:<br>Please define the role and training of the “rehabilitation support worker.” It is unclear whether this role is distinct from the “healthcare support worker” referenced elsewhere. Consistent terminology would improve clarity.	We have revised the manuscript to clarify the role and training of the Rehabilitation Support Worker (RSW) at first mention in the Intervention section and have standardised terminology throughout the manuscript. References to “healthcare support workers” have been corrected to “rehabilitation support workers” to ensure consistency and avoid confusion.
9	Assessment Procedures<br>Baseline assessments were performed either during hospitalization or after discharge, whereas the abstract only mentions assessments after discharge. Please clarify for consistency. In addition, listing the key assessment instruments used (even briefly) would help readers understand feasibility and study burden.	Thank you for this comment. We have revised the Abstract to clarify that baseline assessments were conducted either during hospital admission or post-discharge, ensuring consistency with the Assessments section. We have also updated the outcome measure section to briefly outline the key assessment instruments used, while directing readers to the published protocol for full details.
10	Sample Size Considerations<br>I encourage the authors not to include a sample size calculation as part of the feasibility objective, since preliminary efficacy signals from such a small sample are not meaningful. The initial target enrollment was 60 participants; however, only 19 were enrolled. Please comment on whether this discrepancy itself informs feasibility conclusions.	We agree that preliminary efficacy signals from this small sample are not meaningful. In line with the CONSORT guidance, we have revised this section. Although a target enrolment of 60 was planned, this was not included as a formal progression criterion; in hindsight, a prespecified recruitment target would have been appropriate and shortfall in recruitment does impact feasibility.
11	Feasibility Outcome Tables:<br>Tables 1 and 2 include overlapping information, which may confuse readers. Consider merging or restructuring them. As noted above, please state the rationale for the progression criteria and whether thresholds were defined a priori.	Tables 1 and 2 present related but distinct information (feasibility outcomes vs progression criteria). We have clarified in the Methods/Discussion that progression criteria were defined a priori and provide the rationale for these thresholds. While we could merge the tables or add a progression criteria column to Table 1, we feel this would result in a large and less readable table; Reviewer 1 indicated that the current tables were clear. We are happy to restructure the tables if the editor prefers.
12	CONSORT Flow Diagram<br>Please include detailed reasons for ineligibility, withdrawal, and loss to follow-up.	We have revised the CONSORT flow diagram to include detailed reasons for ineligibility, withdrawal, and loss to follow-up.
13	Citation<br>Reference #30 appears incorrect. Please also cite foundational work that informed the logic model described in the Methods (process evaluation section).	We have checked and corrected Reference #30 and added citations to foundational work that informed the development of the logic model in the method section.

14	Interpretation of Clinical Outcomes<br>The Discussion suggests that participation in the intervention was associated with improvements in DAD scores over 6 months. This change may reflect natural recovery after hospitalization rather than an intervention effect. The authors should avoid causal language and, if possible, cite expected recovery trajectories in similar populations.	We have revised the Discussion to remove causal language and to clarify that changes in DAD scores are descriptive only and cannot be attributed to the intervention in this single-arm feasibility study.
15	Conclusion:<br>The conclusion links high consent rate and participant engagement to potential clinical benefit; these metrics reflect feasibility, not effectiveness, and should not be interpreted as indicators of intervention impact.	Thank you for this comment. We have revised the Conclusions section to ensure that consent rates, engagement, and retention are presented solely as indicators of feasibility and acceptability, and not as evidence of clinical effectiveness. The revised conclusion acknowledges that progression criteria were not fully met and focuses on the modifications required before a definitive RCT could be undertaken.

VERSION 2 - REVIEW

**Reviewer**	**1**
**Name**	**Schimböck, Florian**
**Affiliation**	**Kiel University**
**Date**	**18-Feb-2026**
**COI**	

I have reviewed the revised manuscript and thank the authors for their thorough and thoughtful responses to the reviewers' comments. All points have been addressed satisfactorily, and the revisions have improved the clarity and quality of the manuscript. I have no further comments.

**Reviewer**	**2**
**Name**	**Fuchita, Mikita**
**Affiliation**	**University of Colorado**
**Date**	**02-Mar-2026**
**COI**	

Methods

Under the Eligibility section, please capitalize Confusion Assessment Method in the first sentence.

Methods

Under the Recruitment section, the authors added the following statement in this revision:

"It is important to note that there was variability across the six participating sites in how routinely and systematically delirium screening tools were applied in clinical practice, which influenced the number of patients identified and referred for eligibility assessment."

Inconsistencies in delirium screening are pervasive globally and well-documented in the literature. Given this known challenge, the authors may consider discussing an alternative screening strategy—specifically, the use of trained research personnel to conduct high-fidelity, standardized, twice-daily delirium assessments (which many would consider the minimum standard screening frequency in research and clinical settings). Reliance on routine clinical screening may introduce variability and selection bias, and acknowledging or proposing a more rigorous approach would strengthen the methodological discussion.

Overall Assessment

The authors have done an impressive job revising and substantially improving this manuscript. The conclusions now align clearly with the objective data presented and are appropriately balanced. The discussion articulates future directions with sufficient detail and constructive action steps. In addition, the authors provide a strong contextualization of the trial within the broader literature. The reporting is transparent, thorough, and methodologically rigorous, exemplifying high-quality science.

With the minor revision noted above, this manuscript would merit publication.

VERSION 2 - AUTHOR RESPONSE

	**Reviewer 1**	
	Specific Comments	***Responses***
1	I have reviewed the revised manuscript and thank the authors for their thorough and thoughtful responses to the reviewers' comments. All points have been addressed satisfactorily, and the revisions have improved the clarity and quality of the manuscript. I have no further comments.	Thank you for your positive and supportive comments. We appreciate the time and effort taken to review our manuscript and are pleased that the revisions have improved the clarity and quality of the work.

	**Reviewer 2**	
	Specific Comments	***Responses***
1	Methods<br>Under the Eligibility section, please capitalize Confusion Assessment Method in the first sentence.	We have corrected the text by capitalizing Confusion Assessment Method in the first sentence of the Eligibility section in the Methods

2	Methods<br>Under the Recruitment section, the authors added the following statement in this revision:<br>"It is important to note that there was variability across the six participating sites in how routinely and systematically delirium screening tools were applied in clinical practice, which influenced the number of patients identified and referred for eligibility assessment."<br><br>Inconsistencies in delirium screening are pervasive globally and well-documented in the literature. Given this known challenge, the authors may consider discussing an alternative screening strategy—specifically, the use of trained research personnel to conduct high-fidelity, standardized, twice-daily delirium assessments (which many would consider the minimum standard screening frequency in research and clinical settings). Reliance on routine clinical screening may introduce variability and selection bias, and acknowledging or proposing a more rigorous approach would strengthen the methodological discussion.	Thank you for this suggestion. We agree that reliance on routine clinical delirium screening may introduce variability and potential selection bias. We have expanded the Discussion section to acknowledge this limitation and note that future studies may benefit from incorporating standardised delirium assessments conducted by trained research personnel at regular intervals to improve the consistency and fidelity of delirium detection in research settings.
3	Overall Assessment<br>The authors have done an impressive job revising and substantially improving this manuscript. The conclusions now align clearly with the objective data presented and are appropriately balanced. The discussion articulates future directions with sufficient detail and constructive action steps. In addition, the authors provide a strong contextualization of the trial within the broader literature. The reporting is transparent, thorough, and methodologically rigorous, exemplifying high-quality science.<br><br>With the minor revision noted above, this manuscript would merit publication.	Thank you for your encouraging and thoughtful comments on the revised manuscript. We appreciate the recognition that the revisions have strengthened the clarity, balance, and contextualisation of the study within the existing literature. We have addressed the minor revision noted and hope the manuscript is now suitable for publication.
